# Supplementary material for: LL-37 and citrullinated-LL-37 enhances oxylipins: citrullination attenuates LL-37-mediated COX-2-dependent chemokine response in human bronchial epithelial cells
Source: Respir Res. 2026 Jan 14;27:21. doi: 10.1186/s12931-026-03493-w (PMC12829220; doi:10.1186/s12931-026-03493-w)

**SUPPLEMENTARY INFORMATION**

**LL-37 and citrullinated LL-37 enhances oxylipins: Citrullination attenuates LL-37-mediated COX-2-dependent chemokine response in human bronchial epithelial cells**

Padmanie Ramotar^1^, Mahadevappa Hemshekhar^2,3^, Anthony Altieri^1,4^, Anne M van der Does^5^, Christopher Pascoe^6^ and Neeloffer Mookherjee^1,2,*^

^1^Department of Immunology, University of Manitoba, Winnipeg, MB, Canada.

^2^Manitoba Centre for Proteomics & Systems Biology, Department of Internal Medicine, University of Manitoba, Winnipeg, MB, Canada.

^3^Nursing Department, School of Health and Community Services, Red River College Polytechnic, Winnipeg, MB, Canada.

^4^Department of Immunology, University of Toronto, Toronto, ON, Canada.

^5^PulmoScience Lab, Department of Pulmonology, Leiden University Medical Centre, Leiden, The Netherlands.

^6^Department of Physiology & Pathophysiology, University of Manitoba, Winnipeg, MB, Canada.

*Corresponding Author

Dr. Neeloffer Mookherjee

799 John Buhler Research Centre, 715 McDermot Ave, Winnipeg, MB, R3E3P4, Canada.

Phone : +1 204-272-3115. Email: neeloffer.mookherjee@umanitoba.ca

**Supplementary Figure 1: *Quality control of LL-37 and citLL-37*.** The peptides **(a)** LL-37 and **(b)** citrullinated-LL-37 (citLL-37) were obtained from the company Innovagen (Sweden), and they provided the quality control analyses for the peptides to demonstrate the purity of the peptides synthesized (>95%) and the mass spectrometry analysis.

**(a)**


**(b)**

**Supplementary Figure 2: *Concentration optimization of LL-37 for chemokine responses:*** HBEC-3KT were stimulated with LL-37 or sLL-37 (0.25 and 0.50 µM). Tissue culture (TC) supernatants were collected after 24 h. The abundance of **(a)** IL-8, **(b)** GROα, and **(c)** MIP-3α proteins were measured in TC supernatants by ELISA. Each dot represents an independent experiment (n=5), the box represents the interquartile range with the median line, and the whiskers represent the min-max range. Statistical analysis was performed using Two-Way ANOVA (***p*< 0.001, *****p*< 0.0001, ns=non-significant).


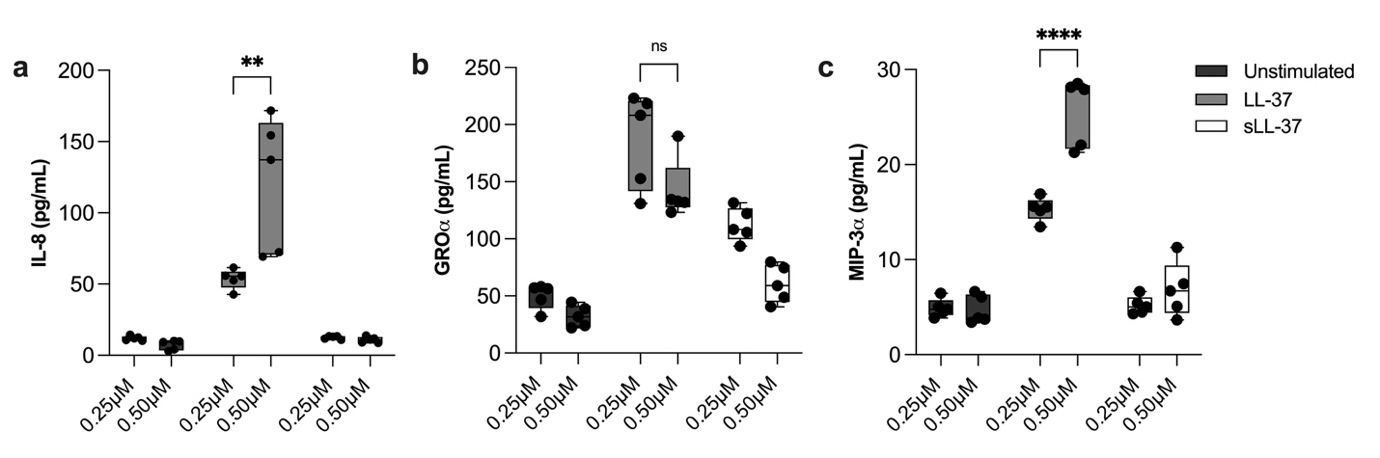


**Supplementary Figure** **3:** ***Optimization of concentration of P2X_7_ inhibitor KN62.*** HBEC-3KT cells were pre-treated with the P2X_7_ inhibitor KN62 (10 nM, 20 nM and 40 nM) for 1 h. Subsequently the cells were stimulated with peptides either LL-37, citLL-37 or sLL-37 (0.50 μM). Tissue culture (TC) supernatants were collected after 24 h. Protein abundance of **(a)** IL-8, **(b)** GROα and **(c)** MIP-3α were measured in TC supernatants by ELISA. Each dot represents an independent experiment (n=4), the bars show IQR with the median line, and the whiskers represent the min-max range. Statistical significance was determined using Two-Way ANOVA (**p*<0.01, ****p*<0.0005 and **** *p*<0.0001)


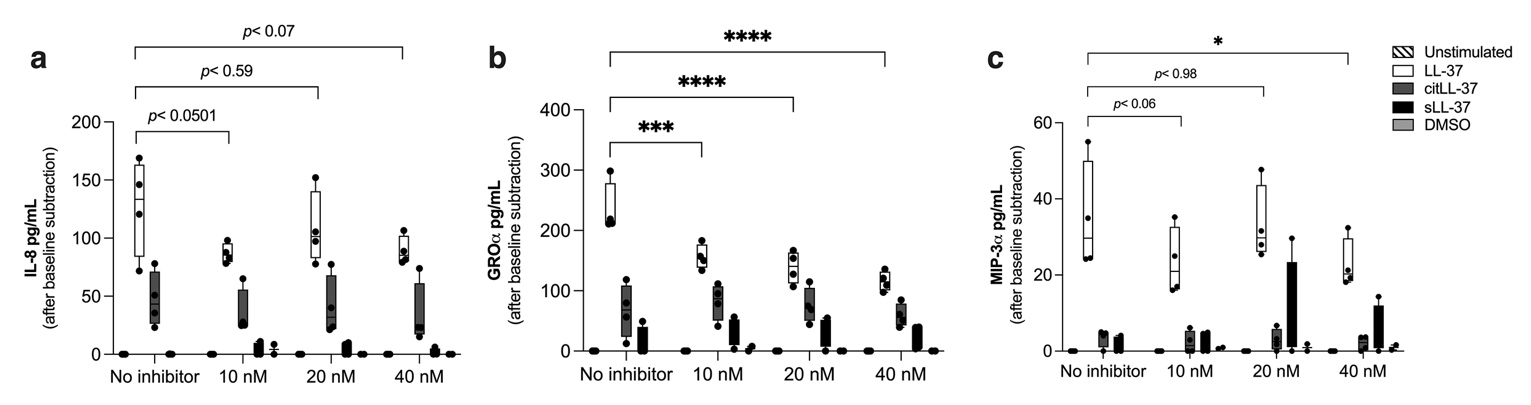


**Supplementary Figure 4:** ***Cytotoxicity assay with the P2X_7_ inhibitor.*** HBEC-3KT cells were pre-treated with P2X_7_ inhibitor KN62 (10 nM, 20 nM, or 40 nM) or the vehicle control (DMSO) for 1 h prior to stimulation with peptides LL-37, citLL-37 or sLL-37 (0.50 μM each). Tissue culture (TC) supernatants were collected after 24 h. Abundance of lactate dehydrogenase (LDH) in the TC supernatant was determined as a marker for cellular cytotoxicity. TC supernatants from cells treated with Triton X100 (1%) were used as 100% cytotoxicity. Each dot represents an independent experiment (n=2), and bars shown the median with minimum-maximum range.

.

**
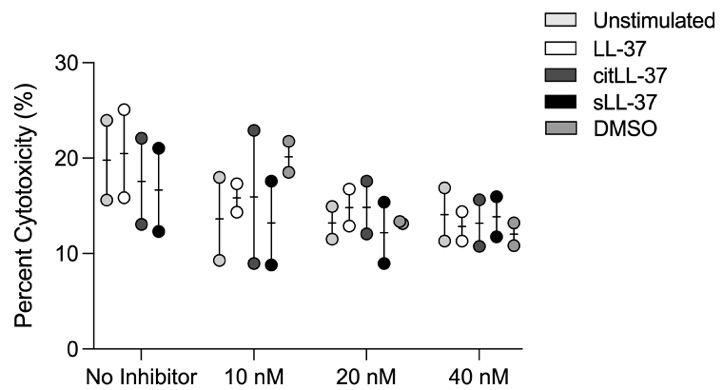
**

**Supplementary Figure 5:** ***LL-37 and citLL-37-mediated enhancement of chemokine transcripts.*** HBEC-3KT cells were stimulated with either LL-37, citLL-37, or sLL-37 (0.50 μM). The mRNA abundance of **(a)** IL-8, **(b)** GROα and **(c)** MIP-3α was examined using qRT-PCR after 4 h. Relative fold changes were calculated compared to unstimulated cells normalized to 1, using the ΔΔCt method after normalization with 18s RNA expression. Results shown are with Log_2_ transformation. Each dot represents an independent experiment (n=4), the bars show IQR with the median line, and the whiskers represent the min-max range. Statistical significance was determined by One-Way ANOVA, # represents statistical significance compared to unstimulated cells (**p*<0.01, ## or ***p*< 0.001, ### or *** *p*<0.0005, and #### or *****p*< 0.0001).

**
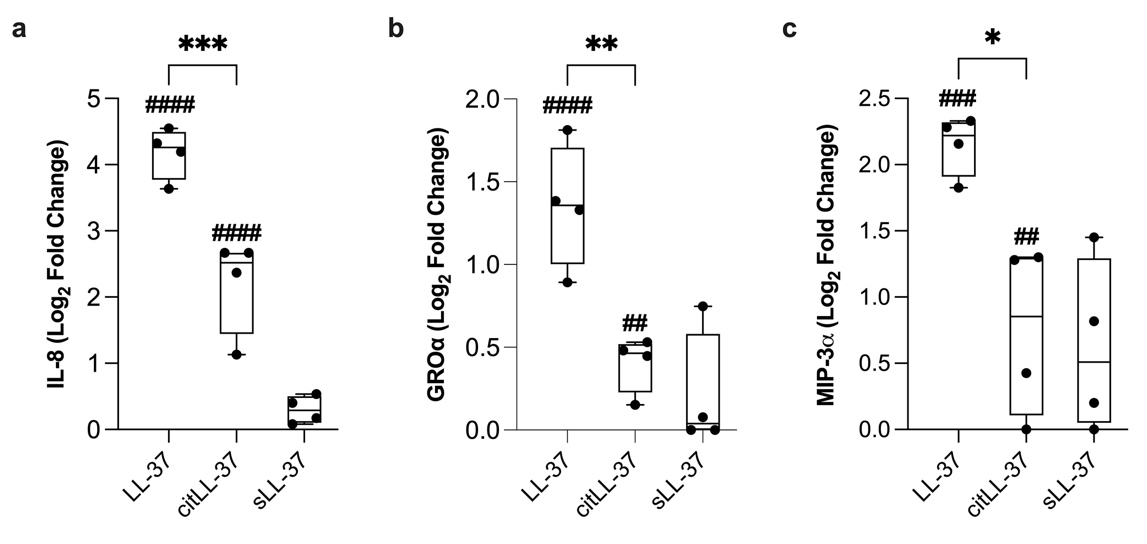
**

**Supplementary Figure** **6: *Optimization of COX-2 inhibitor concentrations.*** HBEC-3KT cells were pre-treated with COX-2 inhibitor Rofecoxib (10 nM, 20 nM or 40 nM) for 1 h. Subsequently the cells were stimulated with either LL-37, citLL-37 or sLL-37 (0.50 μM) for 24 h. Protein abundance of **(a)** IL-8, **(b)** GROα and **(c)** MIP-3α were measured in the tissue culture supernatants by ELISA. Each dot represents an independent experiment (n=2), and bars shown the median with minimum-maximum range (***p*<0.001, ****p*<0.0005 and *****p*<0.0001).


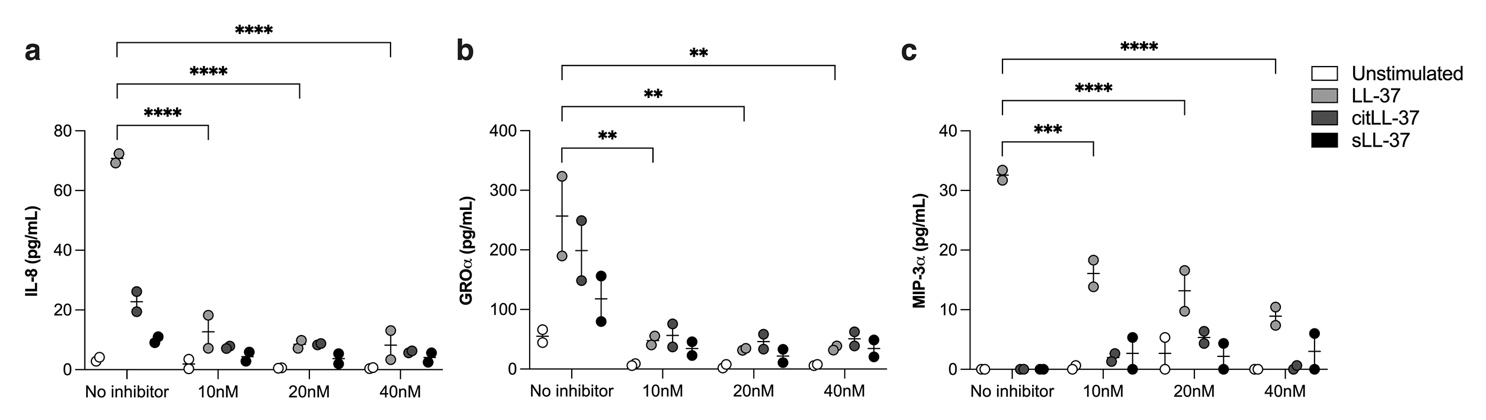


**Supplementary Figure 7: *Cytotoxicity assay with the COX-2 inhibitor.*** HBEC-3KT cells were pre-treated with COX-2 inhibitor Rofecoxib (10 nM, 20 nM or 40 nM) or the vehicle control (DMSO), for 1 h prior to stimulation with peptides LL-37, citLL-37 or sLL-37 (0.5 μM each). Tissue culture (TC) supernatants were collected after 24 h. Abundance of lactate dehydrogenase (LDH) in the TC supernatant was determined as a marker for cellular cytotoxicity. TC supernatants from cells treated with Triton X100 (1%) were used as 100% cytotoxicity. Each dot represents an independent experiment (n=2), and bars shown the median with minimum-maximum range.

.


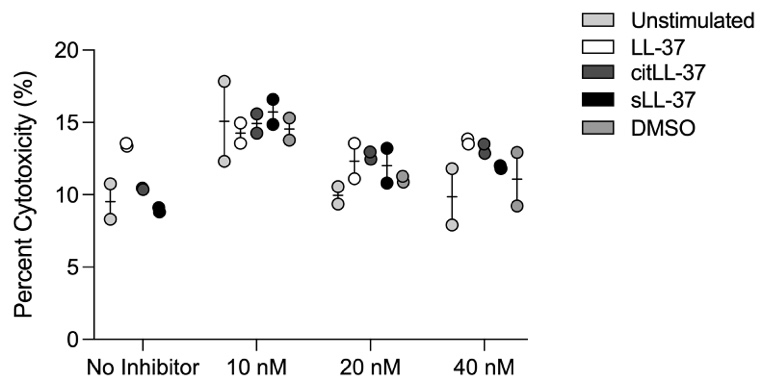


**Supplementary Figure 8:** ***Inhibition of COX-2 suppresses LL-37-mediated chemokine production in human primary bronchial epithelial cells (PBEC)***. Human PBECs were treated with COX-2 inhibitor Rofecoxib (20 nM) for 1 h. Subsequently, the cells were stimulated with either LL-37, citLL-37 or sLL-37 (0.50 μM) for 24 h. The abundance of **(a)** IL-8, **(b)** GROα and **(c)** MIP-3α were examined in the tissue culture supernatants by ELISA. Results shown are after subtracting baseline values obtained in unstimulated cells from each condition, in each independent experiment. Each dot represents an independent experiment, and bars shown the median with minimum-maximum range.

.


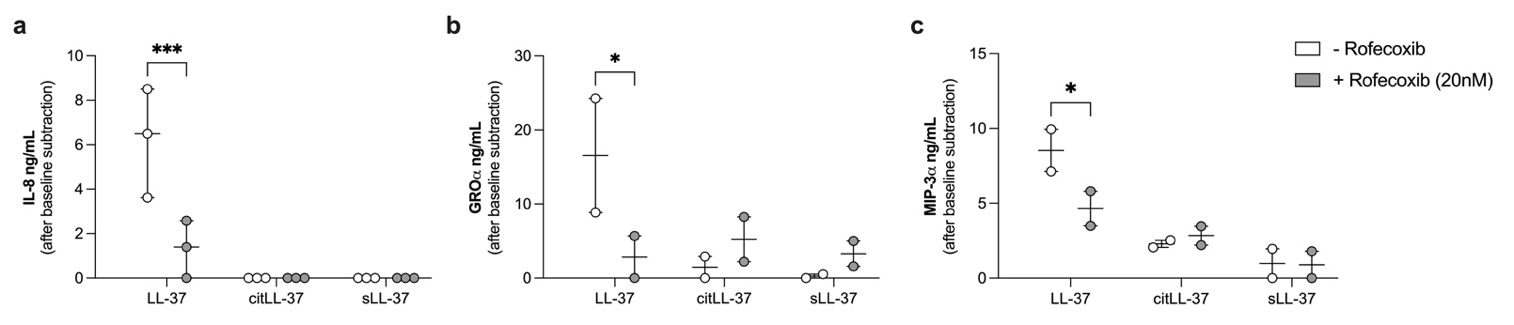


**Supplementary Figure 9: *Cytotoxicity assay with PGE_2_ receptor (EP_1-4_) inhibitors.*** HBEC-3KT cells were pre-treated with PGE_2_ receptor inhibitors (EP_1_; 20 nM, EP_2_; 25 nM, EP_3_; 20 nM) and EP4; 10 nM) or the vehicle control (DMSO), for 1 h prior to stimulation with peptides LL-37, citLL-37 or sLL-37 (0.5 μM each). Tissue culture (TC) supernatants were collected after 24 h. Abundance of lactate dehydrogenase (LDH) in the TC supernatant was determined as a marker for cellular cytotoxicity. TC supernatants from cells treated with Triton X100 (1%) were used as 100% cytotoxicity. Each dot represents an independent experiment (n=2), and bars shown the median with minimum-maximum range.


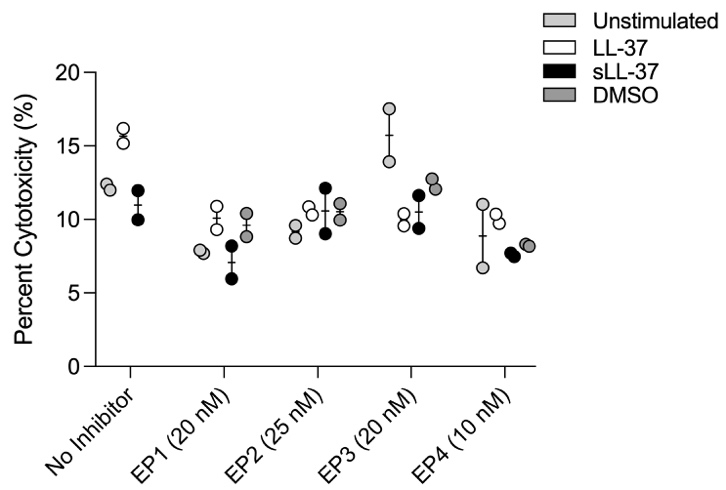

Supplement: Supplementary file 1 — Supplementary Material 1. [file 12931_2026_3493_MOESM1_ESM.docx]
